# An explainable machine learning-based model to predict intensive care unit admission among patients with community-acquired pneumonia and connective tissue disease

**DOI:** 10.1186/s12931-024-02874-3

**Published:** 2024-06-18

**Authors:** Dong Huang, Linjing Gong, Chang Wei, Xinyu Wang, Zongan Liang

**Affiliations:** Department of Respiratory and Critical Care Medicine, West China Hospital, Sichuan University, No. 37 Guoxue Alley, Chengdu, Sichuan 610041 China

**Keywords:** Community-acquired pneumonia, Connective tissue disease, Intensive care unit, Risk factors, Machine learning, Prediction model

## Abstract

**Background:**

There is no individualized prediction model for intensive care unit (ICU) admission on patients with community-acquired pneumonia (CAP) and connective tissue disease (CTD) so far. In this study, we aimed to establish a machine learning-based model for predicting the need for ICU admission among those patients.

**Methods:**

This was a retrospective study on patients admitted into a University Hospital in China between November 2008 and November 2021. Patients were included if they were diagnosed with CAP and CTD during admission and hospitalization. Data related to demographics, CTD types, comorbidities, vital signs and laboratory results during the first 24 h of hospitalization were collected. The baseline variables were screened to identify potential predictors via three methods, including univariate analysis, least absolute shrinkage and selection operator (Lasso) regression and Boruta algorithm. Nine supervised machine learning algorithms were used to build prediction models. We evaluated the performances of differentiation, calibration, and clinical utility of all models to determine the optimal model. The Shapley Additive Explanations (SHAP) and Local Interpretable Model-Agnostic Explanations (LIME) techniques were performed to interpret the optimal model.

**Results:**

The included patients were randomly divided into the training set (1070 patients) and the testing set (459 patients) at a ratio of 70:30. The intersection results of three feature selection approaches yielded 16 predictors. The eXtreme gradient boosting (XGBoost) model achieved the highest area under the receiver operating characteristic curve (AUC) (0.941) and accuracy (0.913) among various models. The calibration curve and decision curve analysis (DCA) both suggested that the XGBoost model outperformed other models. The SHAP summary plots illustrated the top 6 features with the greatest importance, including higher N-terminal pro-B-type natriuretic peptide (NT-proBNP) and C-reactive protein (CRP), lower level of CD4 + T cell, lymphocyte and serum sodium, and positive serum (1,3)-β-D-glucan test (G test).

**Conclusion:**

We successfully developed, evaluated and explained a machine learning-based model for predicting ICU admission in patients with CAP and CTD. The XGBoost model could be clinical referenced after external validation and improvement.

**Supplementary Information:**

The online version contains supplementary material available at 10.1186/s12931-024-02874-3.

## Background

Community-acquired pneumonia (CAP) is an acute lung parenchyma infection caused by bacteria, viruses or fungi acquired outside the hospital. It is one of the most common infectious diseases in clinical practice. Meanwhile, it has been recognized as a major health problem and one of the leading causes of morbidity and mortality in all age groups worldwide [1–3]. Connective tissue disease (CTD) represents a heterogenous group of systemic autoimmune diseases that affect multiple organs, including idiopathic inflammatory myopathies (IIM), rheumatoid arthritis (RA), Sjogren’s syndrome (SS), etc. It is characterized by the presence of circulating autoantibodies and the self-directed chronic inflammation leading to collagen deposition, tissue damage and fibrosis, and ultimately target organs failure. The prevalence and disease burden of CTD continue to increase significantly during past years [4]. Patients with CTD have a higher risk of CAP than the general population according to prior reports [5, 6]. Besides, patients with CTD, especially those with high disease activity, are predisposed to suffer from unfavorable outcomes of CAP compared with those without CTD [7, 8]. Reversely, pneumonia is reported to be the leading cause of intensive care unit (ICU) admission in CTD patients, followed by acute exacerbation of CTD [9]. The underlying mechanisms include immunosuppressive medication use, immune system dysfunction, related comorbidities, etc. Thus, much attention needs to be paid to those patients with CAP and CTD.

It is estimated that 23% of patients who are hospitalized with CAP require ICU admission [10]. Clinical deterioration may occur after hospital admission in CAP patients. However, admission to a non-ICU setting with later transfer to ICU may be associated with poor outcomes and increased mortality [11, 12]. Therefore, apart from timely initiations of appropriate antibiotics and respiratory support, predicting the likelihood of ICU admission is another important issue in the management of CAP patients. Traditional risk score systems such as pneumonia severity index (PSI) and CURB-65 (confusion, uremia, increased respiratory rate, hypotension, and age 65 years or older) have been widely used to facilitate choosing appropriate site-of-care and predicting the prognosis for patients with CAP [13, 14]. Meanwhile, the 2007 Infectious Diseases Society of America / American Thoracic Society criteria for defining severe community-acquired pneumonia (IDSA/ATS 2007 criteria) remains the most pragmatic tool to predict ICU admission in CAP [15]. However, it is reported that the predictive values of PSI and CURB-65 in patients with CAP and CTD were limited [16]. Unfortunately, there are few studies on the individualized risk stratification of them. To our knowledge, there is no specific prediction model for ICU admission on patients with CAP and CTD so far.

Machine learning, a branch of artificial intelligence, can handle plenty of high-dimensional data, analyze complex relationships and identify optimal predictors of clinical outcomes. Over the last few years, the prediction models for both medical diagnosis and prognosis assessment of various diseases have significantly benefited from diverse cutting-edge machine learning algorithms [17, 18]. They are more flexible and may have superior predictive powers than traditional linear models which use variables with statistical significance in some specific diseases according to previous reports [19, 20]. Furthermore, previous evidence demonstrated that machine learning algorithms had good performances in recognizing and predicting the need for intensive care in the initial assessment of patients [21]. In this study, we aimed to establish a machine learning-based model using noninvasive and readily available clinical parameters for predicting the need for ICU admission during hospitalization in patients with CAP and CTD.

## Methods

### Study designs

This was a single-center retrospective observational study on pneumonia patients admitted into West China Hospital of Sichuan University in China between November 2008 and November 2021. The study protocol was approved by the West China Hospital of Sichuan University Biomedical Research Ethics Committee (No.2022 − 733) and was conducted in accordance with the amended Declaration of Helsinki. The requirement for written informed consent from patients was waived due to retrospective design. All personal information of the patients had already been de-identified during the analysis.

### Patients and data

Patients were eligible for inclusion if they were diagnosed with CAP and CTD during admission and hospitalization. The CAP was defined as a new pulmonary infiltrate on chest X-ray or computed tomography (CT) and at least one of the following acute lower respiratory infection symptoms: fever, productive cough, purulent expectoration, dyspnea, pleuritic chest pain, focal chest signs on auscultation, or abnormal peripheral white cell counts [22]. CTD included polymyositis/dermatomyositis (PM/DM), rheumatoid arthritis (RA), Sjogren’s syndrome (SS), systemic sclerosis (SSc), systemic lupus erythematosus (SLE), anti-synthetase syndrome (ASS), undifferentiated connective tissue disease (UCTD) and mixed connective tissue disease (MCTD) in present study. The diagnosis of each type of CTD was established based on corresponding criteria from related clinical guidelines or previous studies [23–28]. Individuals were excluded from the study if they were: (1) under 18 years old; (2) pregnant; (3) having incomplete clinical records. Besides, only the first admission was considered if the patient had multiple admissions during study period.

The clinical data of demographics, CTD types, comorbidities, vital signs and laboratory results during the first 24 h of hospitalization were collected. The first value was used for analysis if any data was repeated. The clinical data were reviewed and collected by two experienced physicians using a standardized data collection form independently. Any disagreement was solved by a third physician or team discussion until a consensus was reached. The primary outcome was the need for ICU admission during hospitalization.

### Feature selection and model construction

**Fig. 1:**
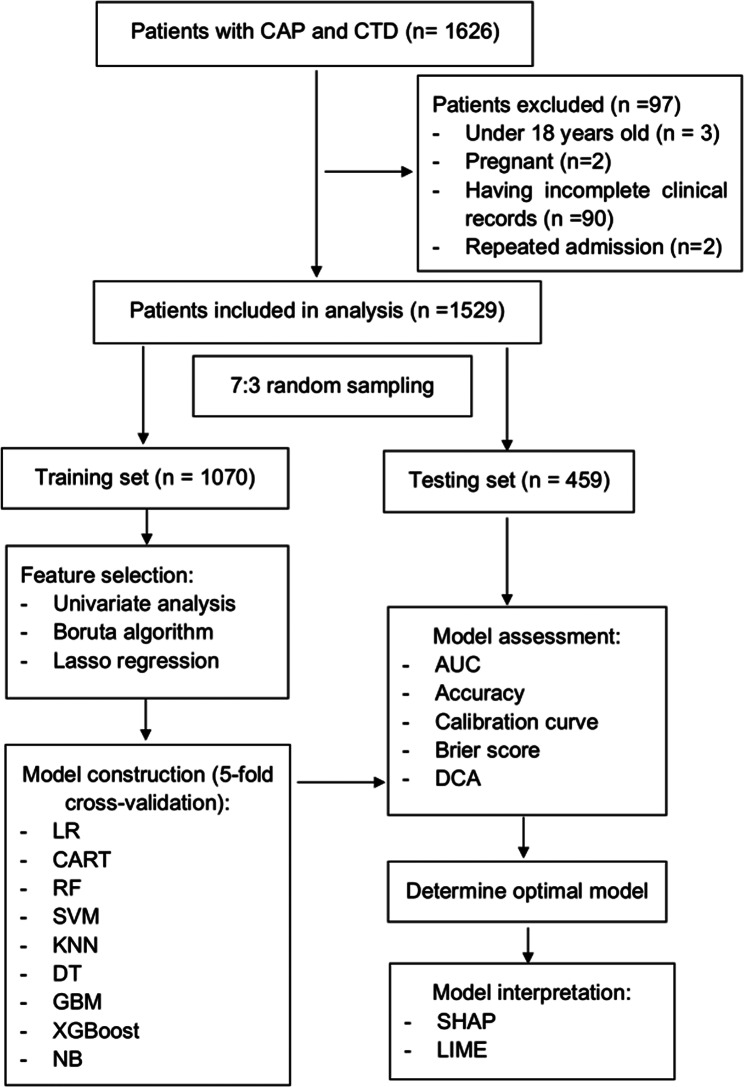
Study flow chart. CAP: community-acquired pneumonia; CTD: connective tissue disease; Lasso: least absolute shrinkage and selection operator; LR: logistic regression; CART: classification and regression tree; RF: random forest; SVM: support vector machine; KNN: k-nearest neighbors; DT: decision tree; GBM: gradient boosting machine; XGBoost: eXtreme gradient boosting; NB: naive bayes; AUC: area under the receiver operating characteristic curve; DCA: decision curve analysis; SHAP: Shapley additive explanations; LIME: Local Interpretable Model-Agnostic Explanations

The flowchart of this study was shown in Fig. 1. The included patients were randomly divided into two sets (70% in training set and 30% in testing set) by simple random sampling. The training set was used for developing the models and the testing set was utilized for evaluating the performances of models.

The baseline variables were screened to identify potential predictors in training set via three independent methods, including univariate analysis, least absolute shrinkage and selection operator (Lasso) regression and Boruta algorithm [29]. The univariate analysis is a classic selection method based on P values. The variables with P value < 0.05 were regarded as statistically significant and were extracted. The Lasso regression model identifies the features having non-zero coefficients as potential predictors. It can eliminate multicollinearity and avoid over-fitting of variables. We used Lasso regression combined with 10-fold cross-validation to analyze the baseline high-dimensional data and screen variables. Boruta algorithm is a feature selection method that depends on the variable importance measure. To be specific, it identifies the most important features by comparing the Z-values of candidate features with that of “shadow features”. The Z-value of each real feature is obtained based on a random forest (RF) classifier in each iteration, and the Z-value of each shadow feature is created by random shuffling of the real features. It can iteratively remove features that have been proved to be less relevant than random shadow features. Thus, only those relevant features with Z-values higher than the maximal Z-value of shadow features by multiple internal bootstraps, are retained. Overlapping variables by intersecting univariate analysis, Lasso and Boruta were obtained to establish prediction models.

Nine supervised machine learning algorithms, including logistic regression (LR), classification and regression tree (CART), RF, support vector machine (SVM), k-nearest neighbors (KNN), decision tree (DT), gradient boosting machine (GBM), eXtreme gradient boosting (XGBoost) and naive bayes (NB), were used to construct prediction models. The 5-fold cross-validation was applied to ensure the stability and accuracy of the models.

### Model assessment

We evaluated the performances of differentiation, calibration and clinical utility of nine models to identify the optimal model. The receiver operating characteristic (ROC) curves were plotted and the areas under the ROC curve (AUCs) were calculated to quantify their discriminative performances. The significant differences of AUCs among models were tested using Delong’s test. Moreover, accuracy, sensitivity, specificity and Kappa value were used as additional descriptions of the predictive abilities of the models [30]. Then, the precision-recall (PR) curves, which plot the positive prediction value (PPV) against the true positive rate (TPR) across all thresholds, were used to further evaluate the discrimination capabilities of the models. Besides, we also calculated the AUCs of three traditional predictive tools, including IDSA/ATS 2007 criteria, PSI and CURB-65, in predicting ICU admission of CAP patients with CTD. The calibration, which represents the agreement between predicted outcomes and actual outcomes, was performed via a bootstrap method with 1000 resamples and assessed by a calibration plot. The decision curve analysis (DCA) based on net benefits at different threshold probabilities was drawn to evaluate the model’s clinical validity and utility.

The performances of the machine learning-based models may be affected by class imbalance due to the low incidence of positive events (ICU admission) in this study. Thus, we performed complementary analyses using up-sampling, down-sampling and synthetic minority oversampling technique (SMOTE) approaches. Sampling techniques are often used to generate balanced datasets (50/50 majority–minority splits) in the training set by up-sampling (over-sampling) or down-sampling (under-sampling). The up-sampling algorithm improves the sensitivity of the minority by synthesizing the minority samples. The down-sampling algorithm achieves the balance of two classes by deleting the majority samples. The SMOTE is an efficient algorithm for addressing class imbalance and reducing over-fitting of the model, employing k-neighbor synthesis to synthesize new minority samples.

### Model interpretation

The Shapley Additive Explanation (SHAP) values of features were evaluated to determine each characteristic’s contribution and significance based on its impact on the final classification outcome. The high SHAP value indicates great impact of a feature on model output. We reported the feature importance for interpreting the optimal model. At last, the Local Interpretable Model-Agnostic Explanations (LIME) technique was performed to further explain the model [31–33].

### Statistical analysis

The clinical characteristics of patients were expressed as the mean ± standard deviation (SD) for continuous variables with normal distribution, the median (interquartile range [IQR]) for continuous variables with non-normal distribution, and the frequency (percentage) for categorical variables. The labels for categorical variables were coded as “1″ for “Yes″ and “0″ for “No″ during statistical analysis. Independent sample t test or Kruskal–Wallis test was used to analyze the differences between the continuous variables as appropriate. The chi-square test or Fisher exact test was performed to analyze the categorical variables. A two-sided *P* < 0.05 was considered statistically significant. We removed variables missing over 30% of observations to ensure the accuracy of study. After that, we employed the multiple imputation method dealing with missing values.

In this study, R software version 4.2.1 (R Foundation for Statistical Computing) was used to implement the statistical analysis. The Lasso and Boruta analyses were performed using the R package “glmnet” and “Boruta”. The R package “caret” was used to train the models. By default, hyperparameter tuning for each model was performed automatically by caret using a standard grid search approach. The SHAP and LIME methods were completed using the “shapviz” and “lime” package.

## Results

### Baseline characteristics

A total of 1626 individuals with CAP and CTD were admitted into our hospital. Among them, 97 individuals were excluded from analysis according to exclusion criteria. The included patients were divided into the training set (1070 patients) and the testing set (459 patients). In the training and testing set, the median age was 56 (IQR: 47, 66) and 57 (IQR:49, 66) years, and 334 (31.2%) and 158 (34.4%) patients were men, respectively. PM/DM was the most common type of CTD (28.9%), and interstitial lung disease (ILD) was observed in 65.1% of all patients. The top 3 comorbidities were hypertension (21.0%), diabetes (12.9%) and congestive heart failure (10.3%). The ICU admission rate and hospital mortality were 292 (27.3%) and 161 (15.0%) in the training set, and 121 (26.4%) and 76 (16.6%) in the testing set. The detailed features were summarized in Table 1, which suggested that the baseline characteristics and clinical outcomes of patients did not differ significantly between two sets roughly.

**Table 1:** Baseline characteristics of patients

Clinical characteristics	Overall (*n* = 1529)	Training set (*n* = 1070)	Testing set (*n* = 459)	*P* value
Demographic characteristics				
Sex: male (%)	492 (32.2)	334 (31.2)	158 (34.4)	0.242
age	56 (47, 66)	56 (47, 66)	57 (49, 66)	0.423
CTD type				
Polymyositis / Dermatomyositis (%)	442 (28.9)	318 (29.7)	124 (27.0)	0.314
Rheumatoid arthritis (%)	380 (24.9)	257 (24.0)	123 (26.8)	0.277
Sjogren syndrome (%)	246 (16.1)	174 (16.3)	72 (15.7)	0.838
Systemic sclerosis (%)	137 (9.0)	96 (9.0)	41 (8.9)	1
Undifferentiated connective tissue disease (%)	98 (6.4)	70 (6.5)	28 (6.1)	0.834
Systemic lupus erythematosus (%)	93 (6.1)	62 (5.8)	31 (6.8)	0.547
Mixed connective tissue disease (%)	89 (5.8)	67 (6.3)	22 (4.8)	0.315
Anti-synthetase syndrome (%)	44 (2.9)	26 (2.4)	18 (3.9)	0.152
Comorbidities				
Interstitial lung disease (%)	996 (65.1)	699 (65.3)	297 (64.7)	0.861
cancer (%)	66 (4.3)	43 (4.0)	23 (5.0)	0.461
chronic liver disease (%)	89 (5.8)	59 (5.5)	30 (6.5)	0.507
congestive heart failure (%)	158 (10.3)	113 (10.6)	45 (9.8)	0.723
cerebrovascular disease (%)	56 (3.7)	40 (3.7)	16 (3.5)	0.926
chronic renal disease (%)	87 (5.7)	54 (5.0)	33 (7.2)	0.124
coronary heart disease (%)	62 (4.1)	45 (4.2)	17 (3.7)	0.753
diabetes (%)	197 (12.9)	140 (13.1)	57 (12.4)	0.785
hypertension (%)	321 (21.0)	225 (21.0)	96 (20.9)	1
COPD (%)	152 (9.9)	107 (10.0)	45 (9.8)	0.981
Vital signs				
Diastolic blood pressure (mmHg)	76 (68, 84)	76 (68, 85)	76 (68, 84)	0.92
Systolic blood pressure (mmHg)	120 (108, 133)	120 (108, 133)	120 (108, 134)	0.949
Respiratory rate (breath/min)	22 (20, 28)	22 (20, 29)	21 (20, 27)	0.208
Heart rate (beat/min)	90 (80, 102)	90 (80, 102)	90 (80, 102)	0.642
Temperature (°C)	36.9 (36.5, 38.6)	36.9 (36.5, 38.6)	37.0 (36.5, 38.8)	0.881
confusion (%)	44 (2.9)	32 (3.0)	12 (2.6)	0.813
Laboratory examinations				
Positive G test (%)	335 (21.9)	229 (21.4)	106 (23.1)	0.506
Positive GM test (%)	41 (2.7)	28 (2.6)	13 (2.8)	0.947
pH	7.41 (7.38, 7.44)	7.41 (7.38, 7.44)	7.41 (7.38, 7.44)	0.158
BUN (mmol/L)	6.8 (4.5, 10.7)	6.9 (4.5, 10.7)	6.5 (4.5, 10.7)	0.677
sodium (mmol/L)	137.3 (133.6, 140.4)	137.5 (133.8, 140.4)	137.0 (133.3, 140.2)	0.324
glucose (mmol/L)	6.93 (4.68, 10.78)	7.15 (4.70, 10.77)	6.01 (4.60, 10.73)	0.118
hematocrit (L/L)	0.36 (0.31, 0.40)	0.36 (0.31, 0.40)	0.36 (0.31, 0.40)	0.62
PF ratio	207 (166, 279)	206 (168, 280)	207 (162, 278)	0.639
hemoglobin (g/L)	115 (103, 128)	115 (102, 127)	114 (103, 128)	0.894
RDW (%)	14.7 (13.7, 16.2)	14.7 (13.8, 16.2)	14.6 (13.5, 16.2)	0.165
platelet (×10 ^9^ /L)	182 (133, 245)	182 (132, 244)	182 (133, 248)	0.681
neutrophil (×10 ^9^ /L)	7.53 (5.02, 11.07)	7.47 (4.94, 11.00)	7.58 (5.28, 11.20)	0.412
lymphocyte (×10 ^9^ /L)	0.98 (0.59, 1.47)	0.99 (0.60, 1.46)	0.98 (0.57, 1.48)	0.79
monocyte (×10 ^9^ /L)	0.33 (0.19, 0.51)	0.33 (0.19, 0.51)	0.33 (0.21, 0.51)	0.411
bilirubin (µmol/L)	9.4 (5.5, 11.9)	9.5 (5.4, 12.1)	9.1 (5.5, 11.8)	0.5
ALT (U/L)	23 (14, 53)	23 (14, 50)	24 (14, 58)	0.206
AST (U/L)	25 (18, 51)	24 (18, 47)	27 (18, 55)	0.11
albumin (g/L)	34.0 (28.7, 39.3)	33.9 (28.6, 38.8)	34.3 (28.8, 40.2)	0.092
globulin (g/L)	28.1 (23.2, 34.7)	28.1 (23.1, 34.9)	28.3 (23.4, 34.1)	0.882
creatinine (µmol/L)	55.00 (44.00, 71.00)	55.85 (43.00, 71.00)	55.00 (44.90, 70.00)	0.957
cystatin C (mg/L)	1.11 (0.94, 1.33)	1.10 (0.94, 1.33)	1.13 (0.95, 1.33)	0.519
triglyceride (mmol/L)	1.39 (1.00, 1.91)	1.37 (1.01, 1.92)	1.44 (0.96, 1.90)	0.978
HDL-C (mmol/L)	1.02 (0.74, 1.36)	1.01 (0.74, 1.33)	1.03 (0.75, 1.41)	0.165
LDL-C (mmol/L)	2.19 (1.56, 2.78)	2.16 (1.55, 2.76)	2.23 (1.60, 2.88)	0.106
creatine kinase (U/L)	52 (26, 154)	50 (26, 146)	56 (28, 169)	0.115
LDH (U/L)	246 (189, 355)	247 (191, 363)	246 (186, 346)	0.451
potassium (mmol/L)	3.50 (3.14, 3.83)	3.50 (3.16, 3.82)	3.44 (3.10, 3.84)	0.206
myoglobin (ng/mL)	43.51 (21.17, 106.60)	42.03 (21.38, 104.05)	47.26 (21.00, 115.68)	0.427
CK-MB (ng/mL)	2.25 (1.09, 4.84)	2.11 (1.06, 4.51)	2.60 (1.22, 5.46)	0.006
NT-proBNP (ng/L)	393 (149, 929)	398 (156, 917)	362 (142, 1003)	0.627
Troponin T (ng/L)	23.0 (11.2, 47.9)	22.1 (11.0, 45.6)	26.2 (11.4, 56.9)	0.061
CRP (mg/L)	29.50 (10.40, 86.00)	30.00 (10.50, 88.75)	27.40 (10.35, 78.25)	0.367
Procalcitonin (ng/mL)	0.09 (0.05, 0.40)	0.09 (0.05, 0.39)	0.09 (0.05, 0.46)	0.366
PT (s)	11.3 (10.4, 12.3)	11.3 (10.5, 12.4)	11.23(10.4, 12.2)	0.099
APTT (s)	27.6 (24.5, 31.3)	27.5 (24.5, 31.5)	27.70(24.5, 30.8)	0.863
fibrinogen (g/L)	3.34 (2.55, 4.22)	3.38 (2.57, 4.27)	3.24 (2.48, 4.15)	0.062
AT III (%)	84.1 (70.4, 100.1)	84.3 (70.0, 99.8)	83.9 (70.7, 100.6)	0.678
D dimer (mg/L)	1.78 (0.74, 4.85)	1.79 (0.75, 4.92)	1.75 (0.72, 4.59)	0.425
PaCO2 (mmHg)	37.4 (32.9, 41.7)	37.4 (32.8, 41.9)	37.4 (33.1, 41.6)	0.663
lactate (mmol/L)	1.61 (1.16, 2.33)	1.61 (1.20, 2.34)	1.60 (1.10, 2.29)	0.193
CD4 + T cell (cell/µL)	346 (195, 516)	344 (198, 529)	349 (194, 499)	0.446
CD8 + T cell (cell/µL)	251 (130, 384)	252 (132, 386)	248 (121, 383)	0.449
pleural effusion (%)	546 (35.7)	386 (36.1)	160 (34.9)	0.692
Outcomes				
ICU admission (%)	413 (27.0)	292 (27.3)	121 (26.4)	0.755
Need for vasopressors (%)	391 (25.6)	274 (25.6)	117 (25.5)	1
Need for IMV (%)	372 (24.3)	264 (24.7)	108 (23.5)	0.68
Hospital mortality (%)	237 (15.5)	161 (15.0)	76 (16.6)	0.502
Hospital LOS (days)	12 (9, 17)	12 (9, 17)	12 (9, 17)	0.314

Data are shown as median with interquartile range (IQR) for continuous variables and number with percentage for categorical variables

CTD: connective tissue disease; COPD: chronic obstructive pulmonary disease; G test: serum (1,3)-β-D-glucan test; GM test: serum Aspergillus galactomannan test; BUN: blood urea nitrogen; PF ratio: the ratio of arterial oxygen partial pressure (mmHg) to fractional inspired oxygen; RDW: red blood cell distribution width; ALT: alanine aminotransferase; AST: aspartate aminotransferase; HDL-C: High density lipoprotein cholesterol; LDL-C: Low density lipoprotein cholesterol; LDH: lactate dehydrogenase; CK-MB: creatine kinase-myoglobin binding; NT-proBNP: N-terminal pro-B-type natriuretic peptide; CRP: C-reactive protein; PT: prothrombin time; APTT: activated partial thromboplastin time; AT III: antithrombin III; ICU: intensive care unit; IMV: invasive mechanical ventilation; LOS: length of stay

### Development of model

**Fig. 2:**
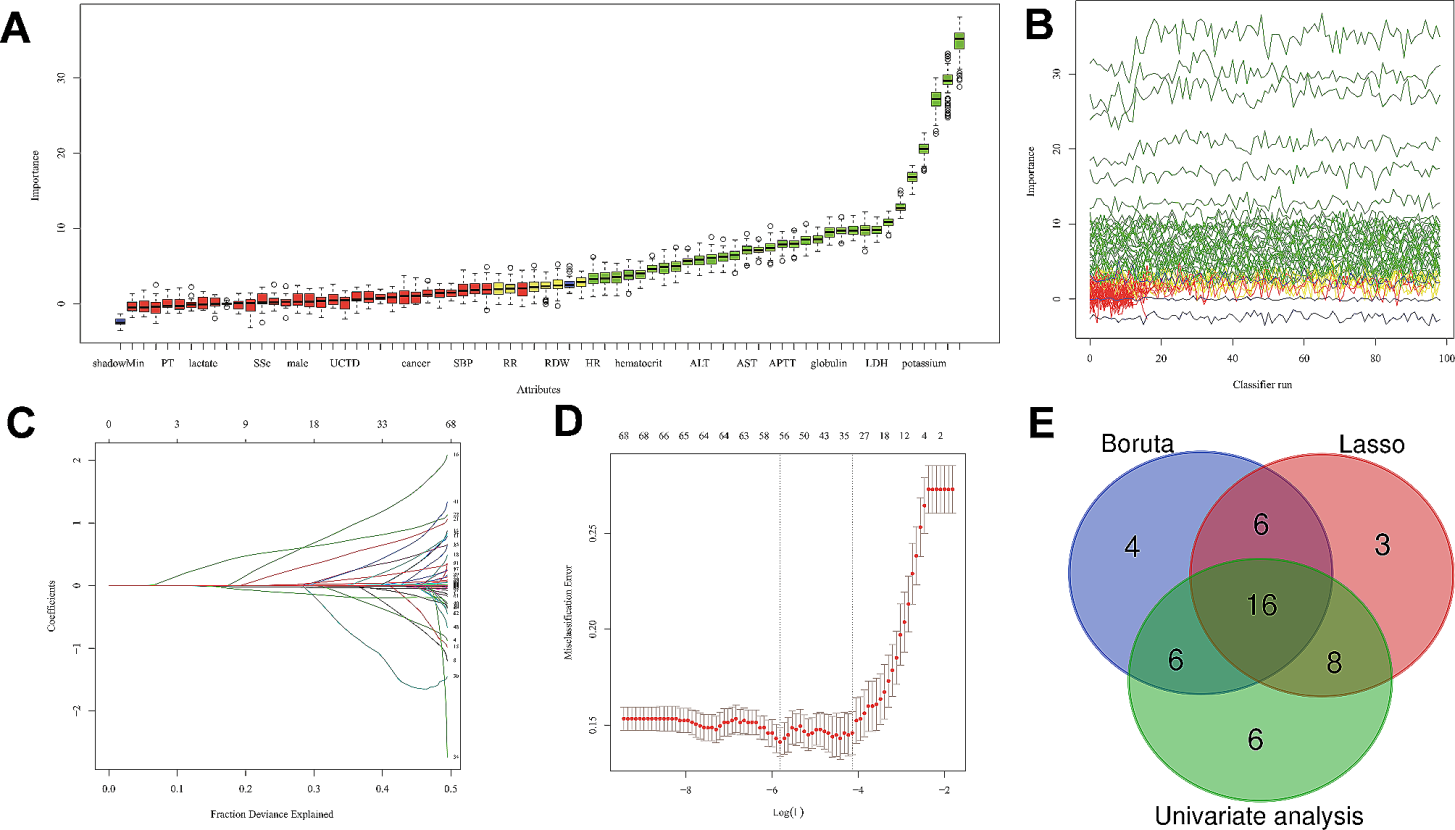
Features selected by Boruta, Lasso and univariate analysis. **A** and **B.** Variables selected by Boruta algorithm. The minimum, average and maximum shadow score are shown in blue. In terms of the score of feature importance, the 32 variables in green are regarded as important variables, while yellow are neutral and red are rejected. **C.** The Lasso regression coefficient profiles of all baseline characteristics. **D.** The optimal lambda selection in the Lasso regression with 10-fold cross-validation. Misclassification errors of different variables against log(lambda) are revealed. The two vertical dashed lines represent the optimal value under the minimum criterion and 1-SE criterion, respectively. The “lambda” is the tuning parameter. A total of 33 predictors with non-zero coefficients are identified. **E.** The Venn diagram of features selected by Boruta, Lasso and univariate analysis. The intersection results of three methods yield 16 clinical characteristics. SE, standard error; Lasso, least absolute shrinkage and selection operator

As shown in Supplementary Table S1, in the univariate analysis 36 variables were significantly different (P values < 0.05) between patients admitted and not admitted into ICU in training set. Among all baseline characteristics, the Boruta algorithm effectively selected 32 potential predictors according to the Z-values (importances) (Fig. 2A and B). Meanwhile, the optimal lambda value was 0.003 by using the Lasso algorithm and 33 variables were selected as potential predictors (Fig. 2C and D). The variables identified by Boruta and Lasso were listed in detail in Supplementary Table S2.

The intersection results of three independent methods were considered to be the optimal features. Thus, a total of 16 clinical characteristics, including N-terminal pro-B-type natriuretic peptide (NT-proBNP), CD4^+^T cell, lymphocyte, C-reactive protein (CRP), positive serum (1,3)-β-D-glucan test (G test), serum sodium, ratio of arterial oxygen partial pressure (mmHg) to fractional inspired oxygen (PF ratio), neutrophil, heart rate (HR), chronic obstructive pulmonary disease (COPD), serum glucose, pH, high density lipoprotein cholesterol (HDL-C), albumin, platelet and confusion, were served as predictors to establish machine learning-based prediction models (Fig. 2E).

### Evaluation of model

**Fig. 3:**
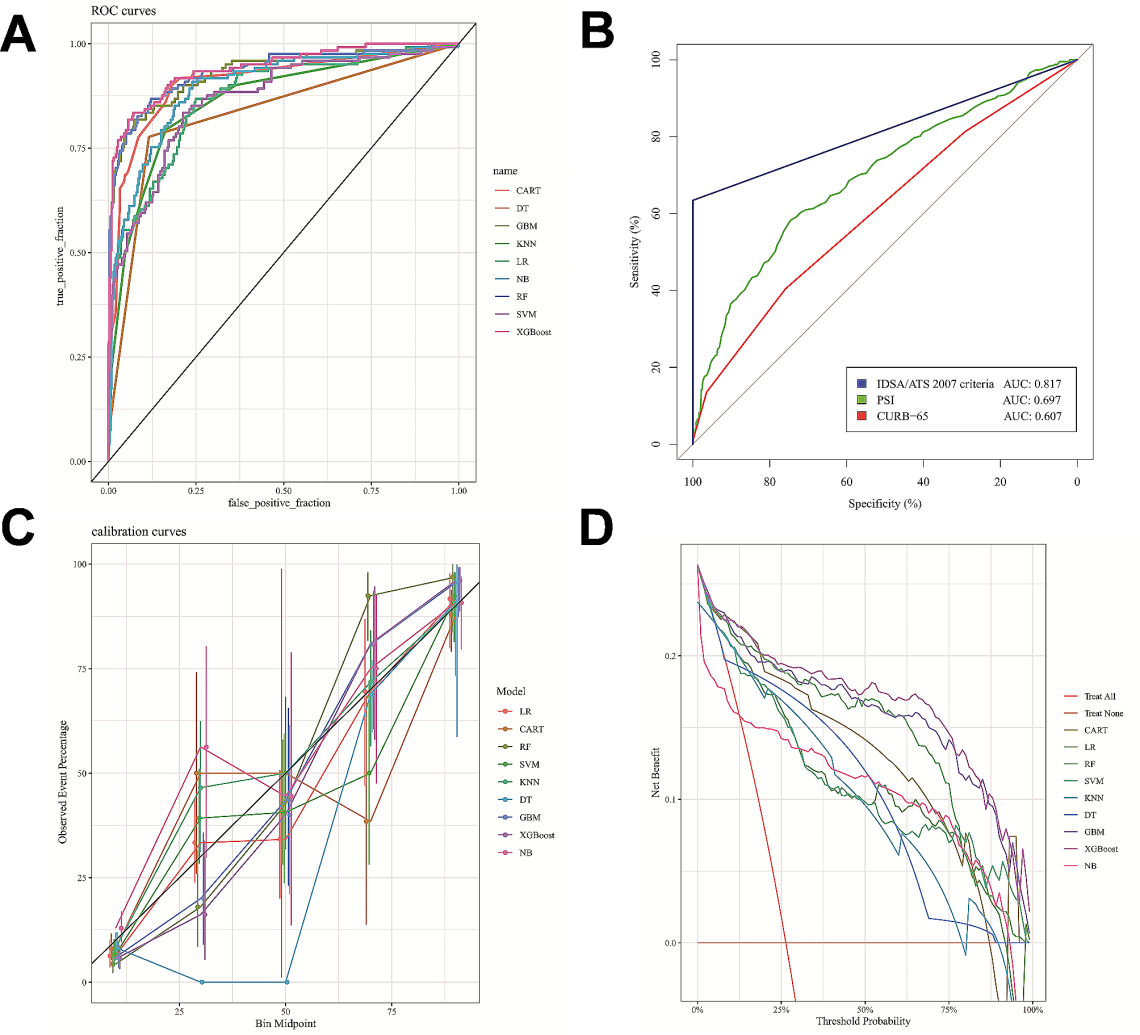
Machine learning-based models used to predict ICU admission in patients with CAP and CTD. **(A)** ROC curves for the machine learning-based models used to predict ICU admission. **(B)** ROC curves for the traditional risk scores used to predict ICU admission. **(C)** Calibration curves of the machine learning-based models. **(D)** DCA of the machine learning-based models. ROC: receiver operating characteristic; DCA: Decision curve analysis; IDSA/ATS 2007 criteria: 2007 Infectious Diseases Society of America / American Thoracic Society criteria for defining severe community-acquired pneumonia; PSI: pneumonia severity index; CURB-65: confusion, uremia, increased respiratory rate, hypotension, and age 65 years or older

All models had accuracy values and AUCs of 0.80 and above in the testing set (Table 2). The ROC curves were shown in Fig. 3A. The XGBoost model achieved the highest AUC (0.941) and accuracy (0.913), suggesting favorable and robust discrimination. LR model was usually used as a traditional baseline model. Thus, the AUCs of other models were compared to that of the XGBoost model and LR model using Delong’s test. The Delong test P value (vs. XGBoost model) were all under 0.05 except for RF model. Meanwhile, the Brier score, Kappa value, sensitivity, specificity, positive predict value and negative predict value of XGBoost model were all superior or similar to that of other models. Furthermore, the XGBoost model also achieved the highest area under the PR curve (0.897) among nine models (Supplementary Figure S1).

**Table 2:** Performance of nine machine learning-based models for predicting ICU admission in the testing set

Model	AUC	Delong test *P* value (vs. LR model	Delong test *P* value (vs. XGBoost model	Accuracy	Kappa value	Sensitivity	Specificity	Positive predict value	Negative predict value	Brier score
LR	0.871	-	< 0.001	0.834	0.537	0.747	0.856	0.562	0.932	0.115
CART	0.911	0.035	0.011	0.878	0.672	0.810	0.898	0.703	0.941	0.093
RF	0.934	< 0.001	0.110	0.909	0.754	0.876	0.918	0.760	0.962	0.082
SVM	0.868	0.711	< 0.001	0.834	0.534	0.753	0.854	0.554	0.935	0.117
KNN	0.865	0.776	< 0.001	0.832	0.553	0.704	0.872	0.628	0.905	0.120
DT	0.834	0.126	< 0.001	0.856	0.641	0.707	0.917	0.777	0.885	0.113
GBM	0.931	< 0.001	0.0298	0.902	0.733	0.880	0.908	0.727	0.965	0.076
XGBoost	0.941	< 0.001	-	0.913	0.767	0.879	0.923	0.777	0.962	0.070
NB	0.896	0.120	< 0.001	0.852	0.568	0.844	0.853	0.537	0.965	0.128

ICU: intensive care unit; AUC: area under the receiver operating characteristic curve; LR: logistic regression; CART: classification and regression tree; RF: random forest; SVM: support vector machine; KNN: k-nearest neighbors; DT: decision tree; GBM: gradient boosting machine; XGBoost: eXtreme gradient boosting; NB: naive bayes

Among the traditional predictive tools, the IDSA/ATS 2007 criteria had higher discriminatory power for ICU admission (AUC: 0.817) compared with PSI (AUC: 0.697) and CURB-65 (AUC: 0.607) (Fig. 3B). However, they did not perform as well as the XGBoost model. The calibration curve and DCA were shown in Fig. 3C and D, both suggesting that the XGBoost model performed best among nine models. Thus, the XGBoost model was considered to be the optimal model. The AUCs of models with up-sampling, down-sampling and SMOTE were found to be similar to the original AUCs in the testing set, as listed in detail in Supplementary Table S3.

### Model interpretation

**Fig. 4:**
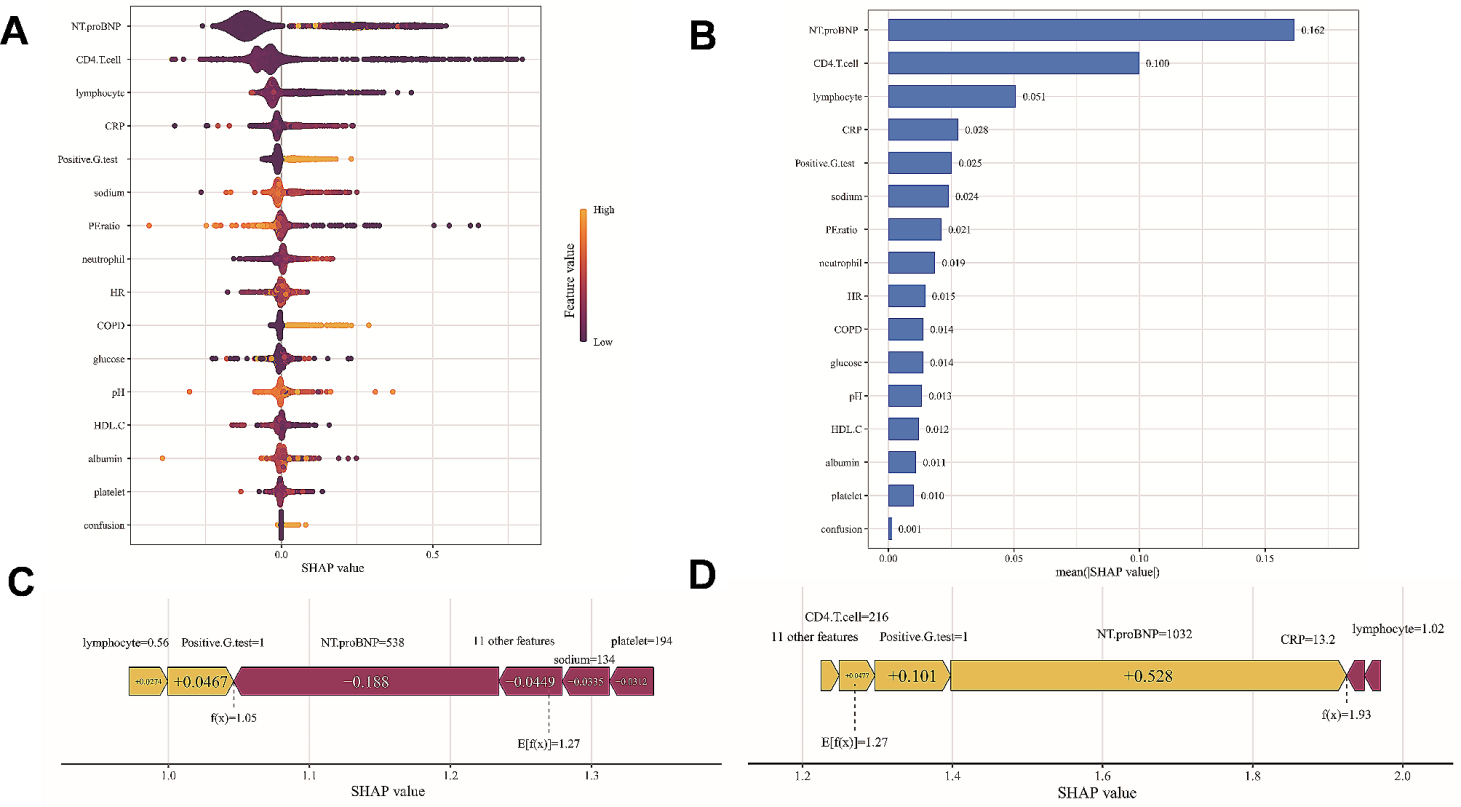
SHAP plots. **(A)** SHAP summary plot shows feature importance for each predictor of the XGBoost model in descending order. The upper predictors are more important to the model’s predictive outcome. A dot is created for each feature attribution value for the XGBoost model of each patient. The further away a dot is from the baseline SHAP value of zero, the stronger it effects the model output. Dots are colored according to the values of features. Yellow represents higher feature values and red represents lower feature values. **(B)** Bar chart of the mean absolute SHAP value for each predictor of the XGBoost model in descending order. **C and D.** The force plots provide personalized feature attributions using two representative examples. C: a patient actually not admitted into ICU; D: a patient actually admitted into ICU. SHAP: Shapley additive explanations; ICU: intensive care unit;

The SHAP values could provide more insights into how the XGBoost model predicted outcomes. The feature importance was summarized by the SHAP summary plot in Fig. 4A. Figure 4B depicted the standard bar chart of the mean absolute SHAP value for each predictor in descending order. The force plots provided personalized feature attributions using two representative examples and illustrated how the SHAP could be used to explain individual model predictions, as shown in Fig. 4C (a patient actually not admitted into ICU) and D (a patient actually admitted into ICU). It started at the base value, that is, the average of all predictions. And then, each input predictor at different level could increase or decrease the predicted probability of outcome. The lengths of arrows reflected the SHAP values for these features. Finally, the predicted output value of model was obtained for a particular patient.

**Fig. 5:**
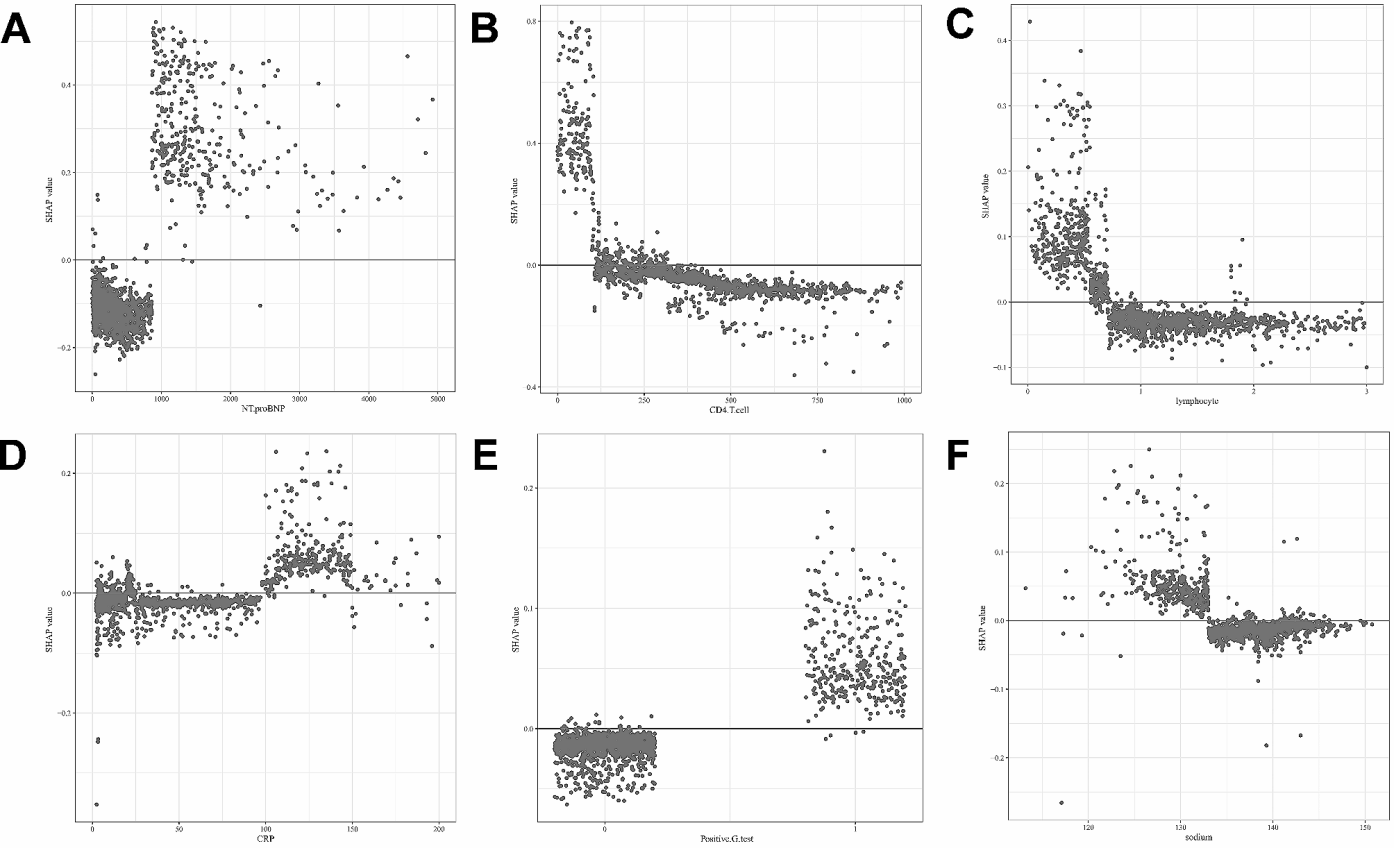
The SHAP dependence plots for the top 6 features with the greatest importance. **A.** N-terminal pro-B-type natriuretic peptide (NT-proBNP). **B.** CD4 + T cell. **C.** lymphocyte. **D.** C-reactive protein (CRP). **E.** positive serum (1,3)-β-D-glucan test (G test). The “1” represents “Yes″ and the “0″ represents “No″. **F.** serum sodium. The SHAP dependence plots show how a single feature affects the output of the XGBoost model. SHAP values for specific features exceed zero, representing an increased risk of ICU admission. SHAP: Shapley additive explanations; ICU: intensive care unit;

We also quantitatively visualized the relationships between main risk factors and outcomes. The SHAP dependence plots illustrated the top 6 features with the greatest importance (Fig. 5A-F). It demonstrated that higher NT-proBNP and CRP values, lower levels of CD4 + T cells, lymphocyte and serum sodium, and positive G test contributed to an elevated risk of ICU admission. Meanwhile, the cutoff value for each variable could also be determined to discriminate between high-risk (SHAP value > 0) and low-risk (SHAP value < 0) of ICU admission.

We selected two random samples from the testing set and used the LIME algorithm to further explain the individual ICU admission forecast. Supplementary Figure S2A depicts a case of patient admitted into ICU. The expected probability of ICU admission was 88% according to the XGBoost model. Supplementary Figure S2B described a case of patient not admitted into ICU. The expected probability of no ICU admission was 60%.

## Discussion

A novel clinically available tool that provides an early assessment and rapid prediction of ICU admission is warranted considering that risk stratification of patients with CAP and CTD remains challenging caused by heterogeneous disease progression. A reliable and accurate prediction model would help clinicians identify specific patients who require special attentions and allocate resources appropriately, which is crucial to timely and effective intervention for prognosis improvement. Machine learning has gained popularity and is increasingly utilized in various domains of biomedicine. To our knowledge, this study is the first to develop a useful machine learning-based model for predicting the risk of ICU admission in patients with CAP and CTD. The promising performance of model was verified by the testing set. The use of advanced machine learning-based models is often limited in clinical practice because of the lack of clear interpretation of their decision-making process. Thus, we used SHAP and LIME to explain what features of the patient are responsible for the given prediction, avoiding the obstacle of “black-box” nature of machine learning algorithms. We found that the NT-proBNP, CD4 + T cell, lymphocyte, CRP, positive G test and serum sodium were the top 6 features of the XGBoost model in terms of their abilities to predict ICU admission.

In our study cohort, the median PSI was only 80 (IQR: 63,103) points, which was slightly lower than that of previous similar studies of CAP patients with a median PSI of about 100 points [34–38]. This may be caused by the differences of included population. The median age, the proportion of male and the number of comorbidities of our cohort were all lower than that of previous cohorts. However, the rate of ICU admission in our study was equal to that in previous reports. Thus, the adverse impact of CTD on the clinical outcomes of CAP should not be ignored. We believed that the CAP patients with CTD might represent a specific subgroup deserving of additional investigations. However, there is still a lack of personalized accurate assessment to guide optimal clinical decisions for them. Li et al. have conducted a similar study including 368 pneumonia patients with CTD treated with glucocorticoids or immunosuppressants [39]. They constructed a prognostic nomogram based on five variables (fever, cyanosis, blood urea nitrogen, ganciclovir treatment and anti-pseudomonas treatment) for predicting the 90-day mortality. The C index of training cohort and validation cohort was 0.808 and 0.762, respectively. Compared to their research, our study had a larger sample size and more predictors. Furthermore, the current study period was more than 10 years with the primary outcome of ICU admission.

The predicting values of these identified predictors are deemed worthy of clinicians’ attention. They can be used to clinically assist physicians to identify high-risk patients at an early stage. The main predictors can be supported by previous studies and theories. NT-proBNP is secreted by the heart in response to excessive stretching of cardiomyocytes. Thus, it is widely used as diagnostic biomarkers for ventricular insufficiency, heart failure and cardiac dysfunction. Previous evidence showed that NT-proBNP was positively correlated with the severity of CAP and remained an independent mortality predictor (hazard ratio [HR]: 1.004, 95% confidence interval [95%CI]: 1.00-1.01) in multivariable analysis [40, 41]. Meanwhile, in patients with CTD, elevated NT-proBNP is considered to be associated with pulmonary arterial hypertension or even heart involvement which may lead to a significant poor prognosis [42–44]. Although treatment with glucocorticoids or immunosuppressive drugs were not recorded, we collected and analyzed the count of lymphocyte subsets instead. It is an objective indicator of the immunosuppressive status of patients. In our study, the CD4 + T cell and lymphocyte were both identified as predictors. This result further confirmed that immunosuppression is indeed a critical indicator of disease severity. Meanwhile, it demonstrated that in various subsets of lymphocyte, low CD4 + T cell was mainly related to ICU admission in patients with CAP and CTD. Wang et al. also demonstrated CD4 + T cells (HR: 0.986, 95%CI: 0.978–0.994), rather than CD8 + T cell, was an independent risk factor for severe CAP in elderly and frailty patients [45]. CRP, a widely utilized inflammatory biomarker, is known to be associated with the severity and mortality of CAP. Meanwhile, it is also a marker for disease activity and risk of death in various types of CTD [46–48]. The G test is a serum pan-fungal marker used to detect the majority of pathogenic fungi, including Aspergillus spp., Candida spp., etc. It is highly accurate for diagnosing invasive fungal infections [49].

However, caution is needed in clinical use of some unexpected results of our study. In disagreement with prior studies, the age and sex, two traditional risk factors, were not identified as predictors in our study. We suspected that, as mentioned above, the median age and the proportion of male in present study were lower compared with that of prior researches, which might be partly responsible for this result. However, these two factors should also be considered by the clinicians, especially when treating those old male patients. Another unexpected result is that, the proportion of ILD was comparable between patients admitted into ICU and those not admitted into ICU. ILD was generally considered as a negative prognostic factor in CAP [50]. However, the studies from Li et al. and Liang et al. also showed that ILD was not independently associated with mortality in pneumonia patients with CTD [5, 39]. More prospective researches are needed to clarify the impact of ILD on CAP patients.

Gearhart et al. have found that assigning differential weights to variables could generate a novel risk score with higher accuracy than original IDSA/ATS 2007 criteria for predicting ICU admission in CAP patients [51]. Consistently, in present study the XGBoost model yielded considerably improved predictions compared with traditional tools. As a highly efficient gradient tree boosting technique, XGBoost is utilized in a variety of medical researches. It can optimally handle diverse variable types and imbalanced datasets, including high-order interactions, non-linearities, discontinuities, etc. Besides, it is resistant to outliers in the predictors and the potential multicollinearity among them. XGBoost uses advanced regularization (L1& L2) to prevent overfitting, optimize prediction model, and increase model’s generalization ability [52–55]. Xu et al. recruited 2302 adults with CAP and found that XGBoost model based on common clinical features had the best performance with AUC of 0.801 in the prediction of ICU admission among various machine learning algorithms [56]. Besides, among patients with COVID-19, XGBoost model was also confirmed to be an excellent prediction model for predicting ICU admission [57, 58].

Our study had some limitations. First, it was a single-center retrospective study, and the selection bias were difficult to completely avoid. Second, this study was only validated using an internal testing set due to the lack of available external validation cohort. The generalizability and robustness of model may be compromised. Third, some data were incomplete because of the retrospective design, including the ILD patterns, such as nonspecific interstitial pneumonia (NSIP), organizing pneumonia (OP) or usual interstitial pneumonia (UIP), the pulmonary function test results, the CTD disease activities, the titers of auto-antibodies, etc. Therefore, they were not included into the analysis. Last, the model was established by baseline characteristics, and the therapies and changes of features after admission were not considered. However, our early investigation may provide a basis for future studies.

## Conclusions

In conclusion, we successfully developed, evaluated and explained a machine learning-based model for predicting ICU admission in patients with CAP and CTD. The XGBoost model showed the optimal performance among nine algorithms. The model could be clinical referenced after external validation and improvement.

### Electronic supplementary material

Below is the link to the electronic supplementary material.

Supplementary Material 1

## Data Availability

The datasets used and/or analyzed during the current study are available from the corresponding author on reasonable request.

## Abbreviations

ICU: Intensive care unit
CAP: Community-acquired pneumonia
CTD: Connective tissue disease
Lasso: Least absolute shrinkage and selection operator
SHAP: Shapley Additive Explanations
LIME: Local Interpretable Model-Agnostic Explanations
ILD: Interstitial lung disease
PSI: Pneumonia severity index
LR: Logistic regression
CART: Classification and regression tree
RF: Random forest
SVM: Support vector machine
KNN: K-nearest neighbors
DT: Decision tree
GBM: Gradient boosting machine
XGBoost: EXtreme gradient boosting
NB: Naive bayes
AUC: Area under the receiver operating characteristic curve
DCA: Decision curve analysis
SMOTE: Synthetic minority oversampling technique
IQR: Interquartile range
NT-proBNP: N-terminal pro-B-type natriuretic peptide
CRP: C-reactive protein
G test: Serum (1,3)-β-D-glucan test
PF ratio: ratio of arterial oxygen partial pressure (mmHg) to fractional inspired oxygen
HR: Heart rate
COPD: Chronic obstructive pulmonary disease
HDL-C: High density lipoprotein cholesterol
PM/DM: Polymyositis / Dermatomyositis
RA: Rheumatoid arthritis
SS: Sjogren’s syndrome
SSc: Systemic sclerosis
SLE: Systemic lupus erythematosus
ASS: Anti-synthetase syndrome
UCTD: Undifferentiated connective tissue disease
MCTD: Mixed connective tissue disease
