# Supplementary material for: An explainable machine learning-based model to predict intensive care unit admission among patients with community-acquired pneumonia and connective tissue disease
Source: Respir Res. 2024 Jun 18;25:246. doi: 10.1186/s12931-024-02874-3 (PMC11186131; doi:10.1186/s12931-024-02874-3)
Supplement: Supplementary file 1 — Supplementary Material 1 [file 12931_2024_2874_MOESM1_ESM.docx]

Table S1. Univariate analysis of 36 potential predictors for ICU admission in training set.

| Variables | Overall (n=1070) | Patients not admitted into ICU (n=778) | Patients admitted into ICU (n=292) | P value |
| --- | --- | --- | --- | --- |
| CTD type |  |  |  |  |
| Polymyositis / Dermatomyositis (%) | 318 (29.7) | 217 (27.9) | 101 (34.6) | 0.039 |
| Sjogren syndrome (%) | 174 (16.3) | 148 (19.0) | 26 (8.9) | <0.001 |
| Comorbidities |  |  |  |  |
| cancer (%) | 43 (4.0) | 25 (3.2) | 18 (6.2) | 0.044 |
| congestive heart failure (%) | 113 (10.6) | 61 (7.8) | 52 (17.8) | <0.001 |
| cerebrovascular disease (%) | 40 (3.7) | 22 (2.8) | 18 (6.2) | 0.017 |
| diabetes (%) | 140 (13.1) | 88 (11.3) | 52 (17.8) | 0.007 |
| COPD (%) | 107 (10.0) | 52 (6.7) | 55 (18.8) | <0.001 |
| Vital signs |  |  |  |  |
| confusion (%) | 32 (3.0) | 7 (0.9) | 25 (8.6) | <0.001 |
| Diastolic blood pressure (mmHg) | 76 (68, 85) | 77 (69, 85) | 74 (66, 83) | 0.006 |
| Systolic blood pressure (mmHg) | 120 (108, 133) | 121 (109, 134) | 116 (105, 130) | 0.004 |
| Respiratory rate (breath/min) | 22 (20, 29) | 21 (20, 28) | 24 (20, 30) | <0.001 |
| Heart rate (beat/min) | 90 (80, 102) | 89 (79, 100) | 97 (85, 109) | <0.001 |
| Temperature (°C) | 36.9 (36.5, 38.6) | 36.9 (36.5, 38.5) | 37.0 (36.5, 38.8) | 0.047 |
| Laboratory examinations |  |  |  |  |
| Positive G test (%) | 229 (21.4) | 109 (14.0) | 120 (41.1) | <0.001 |
| Positive GM test (%) | 28 (2.6) | 8 (1.0) | 20 (6.8) | <0.001 |
| pH | 7.41 (7.38, 7.44) | 7.41 (7.39, 7.44) | 7.40 (7.36, 7.43) | <0.001 |
| BUN (mmol/L) | 6.9 (4.5, 10.7) | 6.6 (4.5, 10.5) | 7.8 (4.6, 11.1) | 0.009 |
| sodium (mmol/L) | 137.5 (133.8, 140.4) | 138.1 (135.2, 140.6) | 134.3 (130.4, 139.0) | <0.001 |
| glucose (mmol/L) | 7.15 (4.70, 10.77) | 5.84 (4.63, 10.70) | 10.00 (5.58, 11.00) | <0.001 |
| hematocrit (L/L) | 0.36 (0.31, 0.40) | 0.37 (0.33, 0.41) | 0.33 (0.27, 0.37) | <0.001 |
| PF ratio | 206 (168, 280) | 210 (174, 311) | 203 (150, 240) | <0.001 |
| hemoglobin (g/L) | 115 (102, 127) | 118 (105, 129) | 107 (100, 118) | <0.001 |
| platelet (×10 ^9^ /L) | 182 (132, 244) | 190 (144, 249) | 162 (115, 218) | <0.001 |
| neutrophil (×10 ^9^ /L) | 7.47 (4.94, 11.00) | 7.02 (4.80, 10.72) | 10.08 (5.43, 11.64) | <0.001 |
| lymphocyte (×10 ^9^ /L) | 0.99 (0.60, 1.46) | 1.09 (0.77, 1.56) | 0.58 (0.30, 0.99) | <0.001 |
| ALT (U/L) | 23 (14, 50) | 21 (13, 44) | 27 (15, 109) | <0.001 |
| AST (U/L) | 24 (18, 47) | 23 (17, 40) | 31 (20, 108) | <0.001 |
| albumin (g/L) | 33.9 (28.6, 38.8) | 35.0 (30.4, 39.4) | 29.5 (25.6, 35.5) | <0.001 |
| HDL C (mmol/L) | 1.01 (0.74, 1.33) | 1.06 (0.78, 1.37) | 0.84 (0.55, 1.18) | <0.001 |
| LDH (U/L) | 247 (191, 363) | 234 (184, 318) | 321 (221, 550) | <0.001 |
| NT-proBNP (ng/L) | 398 (156, 917) | 309 (131, 593) | 1056 (357, 1537) | <0.001 |
| CRP (mg/L) | 30.00 (10.50, 88.75) | 24.50 (8.99, 74.00) | 51.85 (15.17, 121.00) | <0.001 |
| D dimer (mg/L) | 1.79 (0.75, 4.92) | 1.54 (0.62, 3.74) | 7.44 (1.07, 10.45) | <0.001 |
| CD4+ T cell (cell/μL) | 344 (198, 529) | 428 (265, 583) | 153 (72, 301) | <0.001 |
| CD8+ T cell (cell/μL) | 252 (132, 386) | 270 (157, 403) | 204 (100, 339) | <0.001 |
| pleural effusion (%) | 386 (36.1) | 244 (31.4) | 142 (48.6) | <0.001 |

Data are shown as median with interquartile range (IQR) for continuous variables and number with percentage for categorical variables.

ICU: intensive care unit; CTD: connective tissue disease; COPD: chronic obstructive pulmonary disease; G test: serum (1,3)-β-D-glucan test; GM test: serum Aspergillus galactomannan test; BUN: blood urea nitrogen; PF ratio: the ratio of arterial oxygen partial pressure (mmHg) to fractional inspired oxygen; ALT: alanine aminotransferase; AST: aspartate aminotransferase; HDL-C: High density lipoprotein cholesterol; LDH: lactate dehydrogenase; NT-proBNP: N-terminal pro-B-type natriuretic peptide; CRP: C-reactive protein;

Table S2. the predictors identified by Lasso regression and Boruta algorithm.

| Feature selection methods | Features |
| --- | --- |
| Lasso regression | NT-proBNP, CD4+T cell, lymphocyte, CRP, positive G test, sodium, PF ratio, neutrophil, Heart rate, COPD, glucose, pH, HDL-C, albumin, platelet, confusion, PaCO2, monocyte, LDL-C, potassium, AT III, globulin, Sjogren syndrome, cancer, cerebrovascular disease, pleural effusion, Respiratory rate, Hemoglobin, Procalcitonin, Mixed connective tissue disease, congestive heart failure, Diastolic blood pressure, PT |
| Boruta algorithm | NT-proBNP, CD4+T cell, lymphocyte, CRP, positive G test, sodium, PF ratio, neutrophil, HR, COPD, glucose, pH, HDL-C, albumin, platelet, confusion, PaCO2, monocyte, LDL.C, potassium, AT III, globulin, positive GM test, hematocrit, bilirubin, ALT, AST, LDH, myoglobin, APTT, fibrinogen, D-dimer |

NT-proBNP: N-terminal pro-B-type natriuretic peptide; CRP: C-reactive protein; G test: serum (1,3)-β-D-glucan test; PF ratio: the ratio of arterial oxygen partial pressure (mmHg) to fractional inspired oxygen; COPD: chronic obstructive pulmonary disease; HDL-C: High density lipoprotein cholesterol; AT III: antithrombin III; PT: prothrombin time; GM test: serum Aspergillus galactomannan test; ALT: alanine aminotransferase; AST: aspartate aminotransferase; LDH: lactate dehydrogenase; APTT: activated partial thromboplastin time;


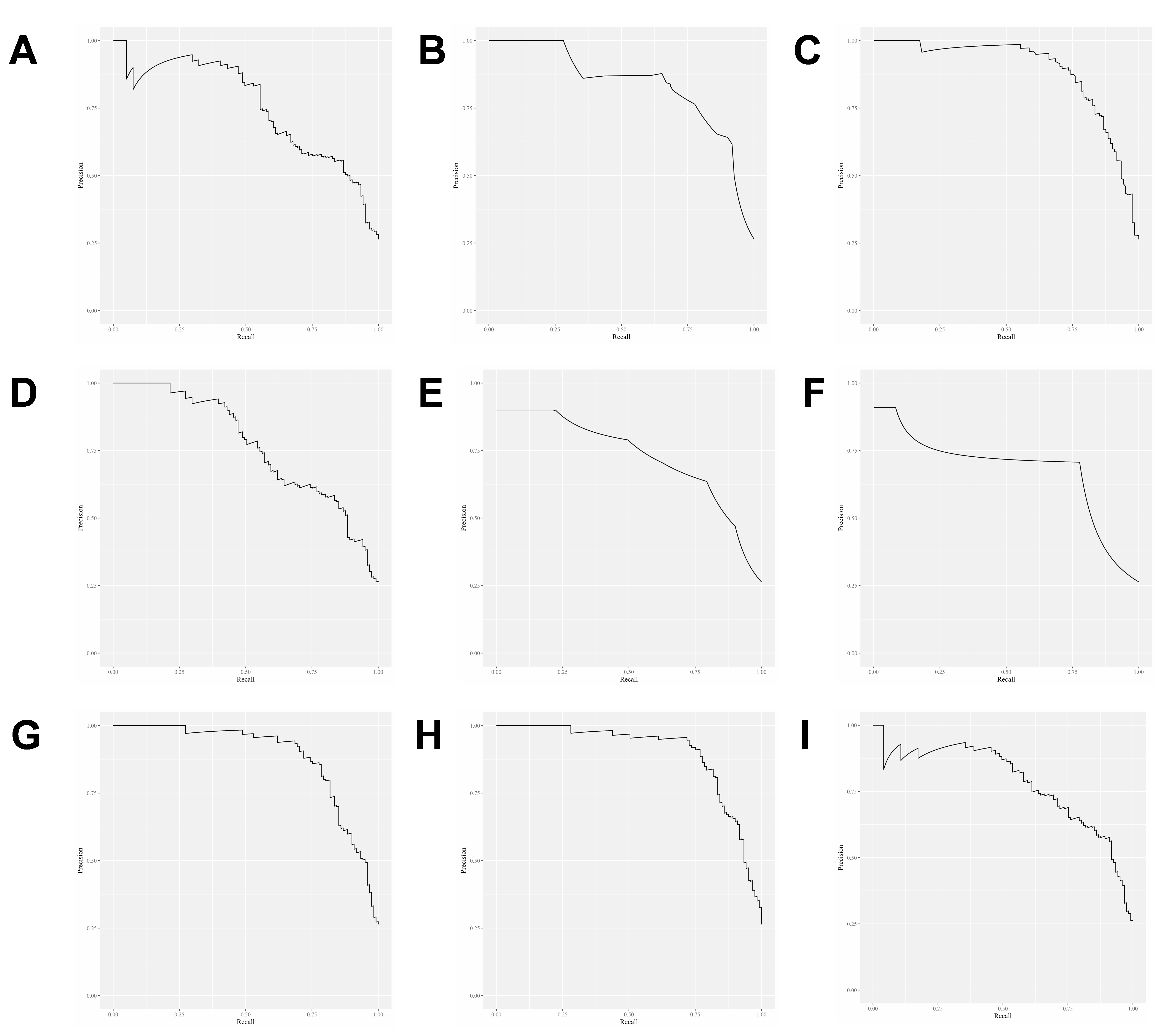


**Figure S1. The precision-recall (PR) curves and the areas under the curves of nine models. A.** LR model (0.751). **B.** CART model (0.834). **C.** RF model (0.887). **D.** SVM model (0.766). **E**.KNN model (0.726). **F**. DT model (0.676). **G**. GBM model (0.885). **H**. XGBoost model (0.897). **I**.NB model (0.783). LR: logistic regression; CART: classification and regression tree; RF: random forest; SVM: support vector machine; KNN: k-nearest neighbors; DT: decision tree; GBM: gradient boosting machine; XGBoost: eXtreme gradient boosting; NB: naive bayes.

Table S3. Comparison of AUCs for machine learning-based models in the testing set using up-sampling, down-sampling and SMOTE.

| Model | Original AUC | AUC of down-sampling model | AUC of up-sampling model | AUC of SMOTE model |
| --- | --- | --- | --- | --- |
| LR | 0.871 | 0.870 | 0.871 | 0.871 |
| CART | 0.911 | 0.879 | 0.876 | 0.864 |
| RF | 0.934 | 0.929 | 0.935 | 0.932 |
| SVM | 0.868 | 0.887 | 0.884 | 0.887 |
| KNN | 0.865 | 0.848 | 0.834 | 0.852 |
| DT | 0.834 | 0.834 | 0.821 | 0.828 |
| GBM | 0.931 | 0.920 | 0.935 | 0.922 |
| XGBoost | 0.941 | 0.922 | 0.921 | 0.937 |
| NB | 0.896 | 0.844 | 0.911 | 0.900 |

AUC: area under the receiver operating characteristic curve; SMOTE: synthetic minority oversampling technique; LR: logistic regression; CART: classification and regression tree; RF: random forest; SVM: support vector machine; KNN: k-nearest neighbors; DT: decision tree; GBM: gradient boosting machine; XGBoost: eXtreme gradient boosting; NB: naive bayes;


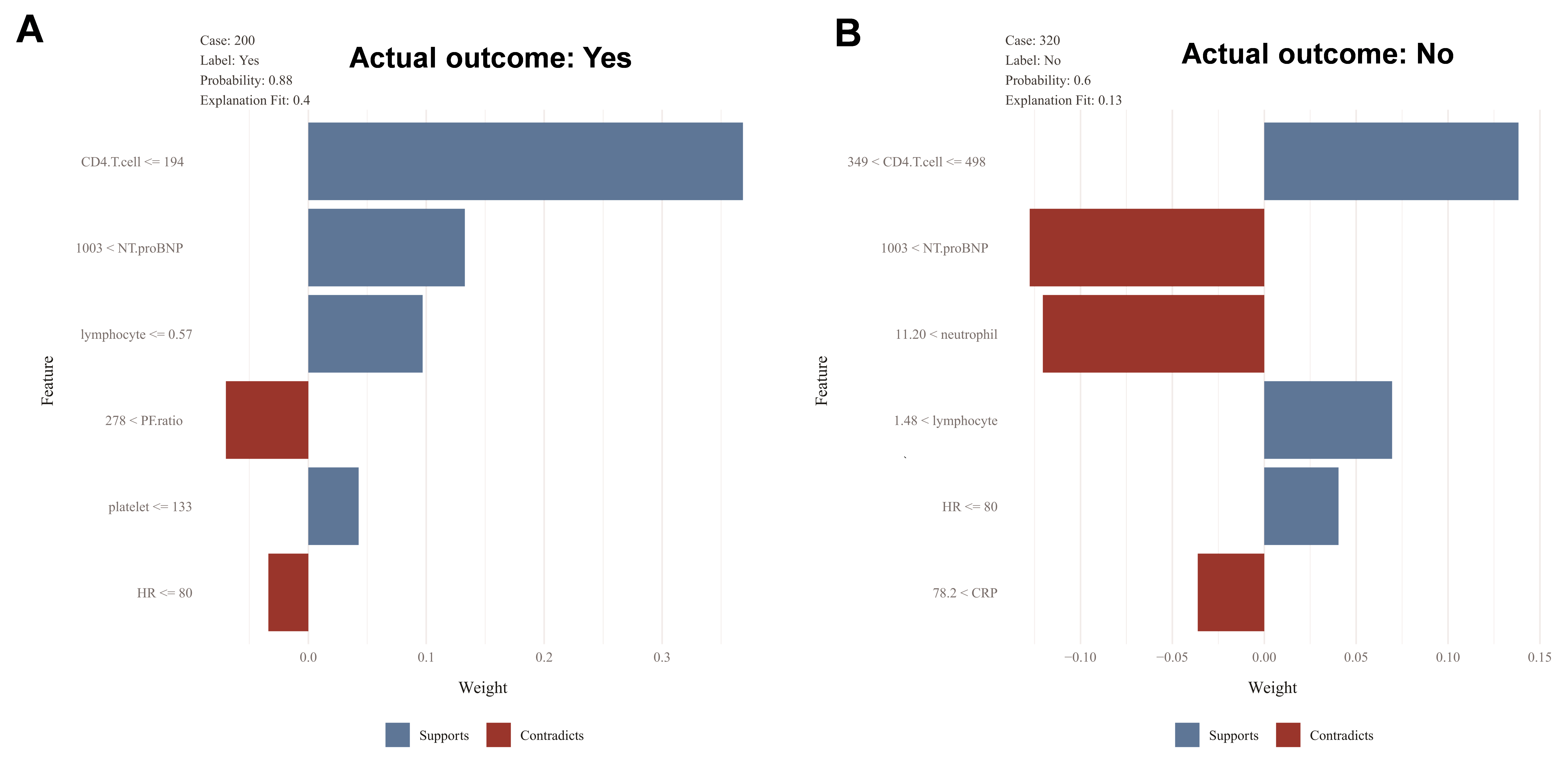


**Figure S2. LIME algorithm for explaining individual’s prediction results.**

**A.** a case of patient actually admitted into ICU (the expected probability of ICU admission was 88% according to the XGBoost model). **B.** a case of patient actually not admitted into ICU (The expected probability of no ICU admission was 60% according to prediction model). It showed the top 6 variables that had the greatest impact on outcomes. The length of the bar for each feature is related to the weight of that feature in the prediction. A longer bar represents a characteristic that contributes more to predict results.
